# Performance of the Adult ADHD Self-Report Scale-v1.1 in Adults with Major Depressive Disorder

**DOI:** 10.3390/bs8040037

**Published:** 2018-03-29

**Authors:** Boadie W. Dunlop, Ruizhe Wu, Kathleen Helms

**Affiliations:** 1 Department of Psychiatry and Behavioral Sciences, School of Medicine, Emory University, 12 Executive Park Drive, 3rd Floor, Atlanta, GA 30329, USA; kat.helms3@gmail.com; 2 School of Public Health, Emory University, Atlanta, GA 30329, USA; ruizhe.wu@emory.edu

**Keywords:** screening, comorbidity, depression, attention, cognitive impairment, anxiety

## Abstract

Attention deficit/hyperactivity disorder (ADHD) is an under-recognized comorbid disorder among patients with mood disorders. ADHD is an independent risk factor for suicidal ideation and behavior and contributes to many aspects of impaired function in adults. Diagnosis of ADHD in Major Depressive Disorder (MDD) patients is challenging due to the overlap in cognitive symptoms between the two disorders. The ADHD Self-Report Scale, version 1.1 (ASRS-v1.1) is a widely used screening instrument for ADHD in adults but its accuracy has not been evaluated previously in treatment-seeking MDD patients. We administered the ASRS-v1.1 to 55 healthy controls and 40 adults with a primary psychiatric diagnosis of MDD who were participating in clinical research studies. ADHD diagnosis was assessed via structured interview with the adult ADHD module of the Mini International Neuropsychiatric Interview Plus version 6.0.0 (MINI) along with a psychiatrist’s assessment. Overall, full-syndrome ADHD was diagnosed in 12.5% of the MDD patients. MDD patients endorsed all 18 items of the ASRS-v1.1 more frequently than the healthy controls and the number of ASRS-v1.1 items endorsed correlated with levels of anxiety in the MDD patients. The ASRS-v1.1 demonstrated fair performance for identifying full syndrome DSM-IV ADHD diagnosis, with sensitivity 60%, specificity: 68.6%, positive predictive value 21.4%, negative predictive value 92.3% and total classification accuracy of 67.5%. Positive predictive value improved substantially when the ADHD criterion requiring symptom onset before age 7 was omitted. In adult MDD patients, a negative ASRS-v1.1 screen strongly suggests the absence of ADHD but positive screen results require careful evaluation to determine whether self-reported ADHD symptoms simply emerge from depression or whether comorbid ADHD is present.

## 1. Introduction

Attention deficit/hyperactivity disorder (ADHD) is a neurodevelopmental disorder characterized by hyperactivity, inattentiveness and impulsivity that onsets during childhood [1]. Although symptoms may diminish in adulthood, the full syndrome commonly persists [2], afflicting an estimated 1.2–7.3% of adults around the world [3], including 4.4% of adults in the United States [4]. Individuals with ADHD experience substantially greater financial burdens, more accidents, incur more health care costs and engage in more criminal behaviors than people without a history of ADHD [5,6]. ADHD can be classified as a primarily inattentive type, primarily hyperactive-impulsive type, or a combined type when criteria for both types are met. Inattentive symptoms, such as daydreaming and forgetfulness, are particularly detrimental to maintaining satisfactory work productivity [7].

Adults with ADHD have substantially (2–3 fold) higher rates of having mood, anxiety, or substance use disorders, as established by both epidemiological [3,8] and clinical studies [9,10]. However, the reciprocal relationships (i.e., the proportion of adults with specific psychiatric disorders who also have ADHD) has been less frequently examined. In the National Comorbidity Survey Revised, a community household survey, the prevalence of ADHD among adults with Major Depressive Disorder (MDD) was 9.4%. Similar rates of ADHD were found among adults with any anxiety disorder (9.5%) or any substance use disorder (10.4%), all of which were roughly half the rate of 21% among adults with bipolar disorder [8].

The limited data about ADHD among patients with depressive disorders represents a particularly important knowledge gap. The clinical significance of identifying ADHD comorbidity among patients with depression is evident in the greater levels of functional impairment, longer depressive episodes and the increased suicidal ideation and behavior among comorbid patients than those with MDD without ADHD [11,12]. Despite the demonstrated importance of ADHD comorbidity, only two published studies have examined the prevalence of ADHD in a psychiatric setting among adults with MDD in a major depressive episode, finding a 7.6% [13] to 14% [9] prevalence. The symptoms of ADHD, particularly those related to inattention, are frequently experienced by patients with mood, anxiety, or substance use disorders, even among patients without comorbid ADHD, which complicates diagnostic efforts. Due to the symptomatic overlap of ADHD with other disorders, particularly cognitive symptoms but also irritability, anxiety, psychomotor agitation and sleep problems [14], many clinicians struggle to detect, diagnose and treat ADHD when present [15,16]. Conversely, limitations in time or expertise may hinder clinicians’ efforts to rule out a diagnosis of ADHD when symptoms of inattention, hyperactivity, or impulsivity are better explained by another psychiatric condition.

A widely used screening instrument for identifying adult ADHD is the Adult ADHD Self-Report Scale, version 1.1 (ASRS-v1.1). Using a total score of ≥4 on the six-item Part A section of the scale as the threshold to indicate a positive screening test, the developers of the ASRS-v1.1 reported moderate sensitivity (68.7%), along with excellent specificity (99.5%) and total classification accuracy (97.9%) when assessing symptoms among a community sample [17]. Despite performing well as a screening instrument in general adult populations, the ASRS-v1.1’s accuracy in comorbid populations is more inconsistent [18,19]. Although it is crucial to accurately identify ADHD when it is present in patients with MDD, there are important iatrogenic risks that arise from over-diagnosis, such as inappropriate prescribing of stimulant medications, which may induce addiction and adversely impact cardiovascular health [20]. Consequently, there is great clinical relevance for fully understanding the utility of ADHD screening in patients with primary psychiatric disorders such as MDD.

Remarkably, there are no published studies on the accuracy of the ASRS-v1.1 among adults with MDD. Here we report an evaluation of the ASRS-v1.1 among a sample of adult outpatients with MDD participating in clinical research studies. We hypothesized that the ASRS-v1.1 would have high sensitivity and negative predictive value but low positive predictive value, due to the expectation that depressed patients would frequently endorse the inattentive and procrastination items on part A of the instrument.

## 2. Materials and Methods

The data being analyzed for this paper was collected from two studies of MDD that enrolled adult healthy control subjects and MDD patients through the Mood and Anxiety Disorders Program at Emory University in Atlanta, Georgia, between August 2013 and September 2016. Both studies compared healthy control (HC) adults with those with MDD. One study examined a blood test for MDD (data not published); the other examined differences in decision-making between adults with and without MDD (clinicaltrials.gov NCT01916824) [21]. The institutional review board of the Emory University approved both studies. Written informed consent was obtained from all participants and all data were de-identified.

### 2.1. Participants

The psychiatric assessment procedure for both studies was identical (described below) and both studies enrolled adult men and women, ages 18–65 using similar inclusion and exclusion criteria. Eligibility criteria for HC included absence in the past year of a DSM-IV mental illness diagnosis (including ADHD), no history of MDD or dysthymia, no history of psychotropic medication use and a score of ≤7 on the 17-item Hamilton Depression Rating Scale (HDRS) [22]. Depressed patients had to meet a primary DSM-IV psychiatric diagnosis of non-psychotic MDD, score ≥ 15 on the HDRS and be off all psychiatric medications (other than sedative/hypnotic) for one month prior to assessment. For all participants, exclusion criteria included meeting DSM-IV criteria for a lifetime diagnosis of bipolar disorder, a primary psychotic disorder (e.g., schizophrenia), or dementia, the presence of any unstable or central nervous system or other medical illness that would interfere with cognition or participation, or presenting with a level of suicide risk, as evaluated by a study physician, that required urgent intervention.

### 2.2. Measures

All participants were assessed with the Structured Interview for DSM-IV (SCID) [23] and the Mini International Neuropsychiatric Interview Plus version 6.0.0 (MINI) Adult ADHD Module (module TA) [24]. This module of the MINI is divided into three sections: TA1, comprised of the 9 DSM-IV ADHD inattention criteria; TA2, comprised of the 9 DSM-IV ADHD hyperactivity/impulsivity criteria; TA3, a question whether the symptoms from TA1 or TA2 began before the age of 7; and TA4, a question whether the symptoms caused functional problems at work, school, home, or with family or friends. For the current analysis, if six or more items from section TA1 or section TA2 were endorsed, *symptom count criteria* for ADHD were considered met. To achieve the *formal diagnosis* of DSM-IV adult ADHD, participants also had to be scored positively on both sections TA3 and TA4 and have the diagnosis confirmed by a study psychiatrist. To confirm the diagnoses of ADHD, the study psychiatrist assessed the temporal course of ADHD symptoms and established that the symptoms of ADHD had emerged prior to the first lifetime depressive episode and could not be better explained by another psychiatric diagnosis (DSM-IV Criterion E), including posttraumatic stress disorder arising from traumatic events experienced in childhood. The SCID was used to confirm the diagnosis of MDD among depressed participants and to identify comorbid psychiatric disorders.

The ASRS-v1.1 is a self-report form used to assess symptoms of ADHD based on the 18 DSM-IV symptom criteria [17]. The instrument is comprised of two parts: Part A (6 questions) and Part B (12 questions). For each item, respondents are asked to indicate how often the stated symptom occurred over the prior six months, with five options: never, rarely, sometimes, often, or very often. For all 18 items, responses of “often” or “very often” are considered positive (indicated by shaded boxes on the questionnaire). In addition, for items 1, 2, 3, 9, 12, 16 and 18, a response of “sometimes” is also scored positive. A patient is considered to screen positive on the ASRS-v1.1 if they endorse 4 or more of the Part A questions at these threshold levels. Although Part B is not used for diagnostic purposes, these items provide insight into the frequency of symptoms and can be used to help elicit other the symptoms the patient may suffer from [15].

To assess depression severity, the HDRS was administered and a study physician scored the Clinical Global Impression-Severity (CGI-S) [25]. The Hamilton Anxiety Rating Scale (HAMA) [26] was used to assess severity of anxiety symptoms. To examine the level of rumination over negative cognitions, the self-report Ruminative Responses Scale (RRS) [27] was completed. The RRS is a self-report questionnaire that assesses the frequency of rumination using a 1 (almost never) to 4 (almost always) scale for 22 items. Higher scores on all three of these scales reflect greater severity of the symptoms. The Childhood Trauma Questionnaire (CTQ) [28], a 28-item self-report instrument, was used as a measure of adverse childhood experiences. The CTQ asks about five kinds of childhood maltreatment, scoring each item from 0 to 5 (never true to very often true). Self-report assessments were administered after the clinician-rated measures.

### 2.3. Statistical Analysis

Consistent with the standard screening threshold for the ASRS-v1.1, participants who scored ≥4 on Part A of the questionnaire were considered to screen positive for ADHD. The gold standard for *formal ADHD diagnosis* was meeting all DSM-IV diagnostic criteria for ADHD as assessed by module TA of the MINI, along with confirmation of the diagnosis by a study psychiatrist’s interview. There has been growing concern that the DSM-IV requirement that ADHD symptoms be present before the age of 7 excludes many afflicted individuals [29], which led to the diagnostic revision in DSM-5 that symptoms should be present before the age of 12 [1]. Consequently, to more broadly examine ADHD symptoms among participants who may have been excluded due to the DSM-IV age 7 criterion, we also classified any participant who endorsed six or more items on section TA1 or TA2 of the MINI as meeting the *symptom count criterion* for ADHD.

Mean scores on the study measures were calculated and compared using independent sample *t*-tests to compare those meeting the case definitions of ADHD and separately for those meeting the ASRS-v1.1 positive screening threshold. To control for multiple comparisons across the ASRS-v1.1 items, we applied a Bonferroni correction, requiring a *p*-value of 0.00278 (0.05/18) to indicate statistical significance. We also evaluated categorical associations with these measures using Chi Square tests, applying Fisher’s exact test when cell sizes had expected values <5.

To assess the operating characteristics of the ASRS-v1.1 in our sample, two-way tables were constructed to calculate the sensitivity, specificity, positive predictive value (PPV) and negative predictive value (NPV) and accuracy of the ASRS-v1.1. These measures were calculated separately for two different case definitions: (1) those who met the symptom count criteria definition on the MINI and (2) those who met the full formal DSM-IV diagnostic criteria.

## 3. Results

### 3.1. Participants

There were 40 MDD and 55 HC participants across the two studies with complete MINI and ASRS-v1.1 data. Demographic characteristics were similar across the two groups. The HC sample had a mean age of 44.0 ± 11.5 years, was 70.9% female and had a race distribution of 50.9% African-American, 29.1% white and 20% multiple or another race. The MDD sample had a mean age of 49.5 ± 8.1, was 72.5% female, with a race distribution of 32.5% African-American, 47.5% white and 20% multiple or another race.

### 3.2. ASRS-v1.1 Descriptive Results

**Figure 1:**
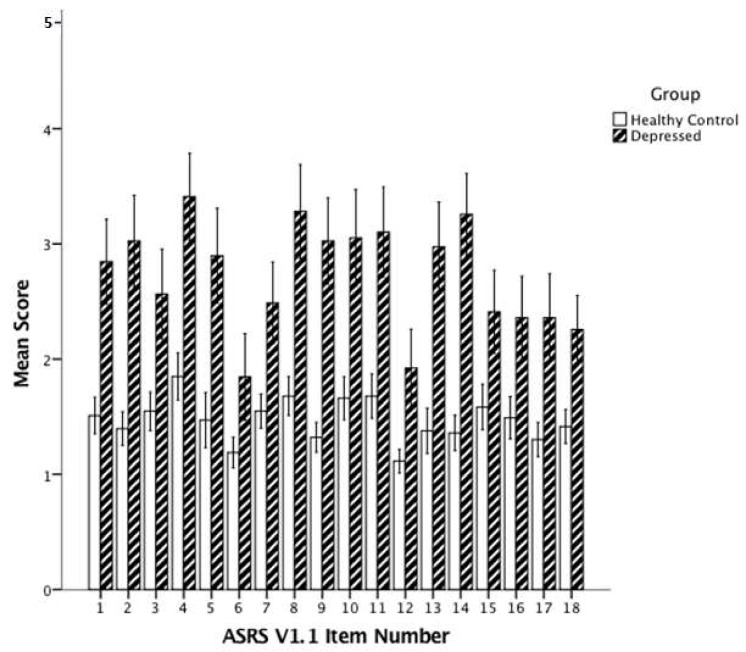
Mean scores for the 18 ASRS-v1.1 items in healthy control and depressed patients. ASRS-v1.1: Adult ADHD Self-Report Scale Symptom Checklist. Note: To quantify responses, we assigned a numerical score to each of the five response options: “never” = 1; “rarely” = 2; “sometimes” = 3; “often” = 4; “very often” = 5. All *p* < 0.005. Error bars: 95% CI.

Figure 1 shows a clustered histogram of the ASRS-v1.1 items between the MDD and HC participants. MDD participants scored significantly higher on all 18 items (all *p* < 0.00278). Because the ASRS-v1.1 is a screening tool to identify people who endorse the items at a frequency that suggests an ADHD diagnosis, we also examined the proportion of subjects endorsing each item at a level qualifying as a significant symptom (i.e., in the shaded box on the questionnaire).

As shown in Table 1, MDD participants endorsed many of the ASRS-v1.1 items at a high frequency. In particular, the items regarding difficulties with wrapping up details of projects, listening when being spoken to directly, or getting organized for a project were all endorsed at the threshold level by ≥60% of the MDD participants. Indeed, four of the six Part A items that are used as the screen for ADHD were endorsed at the threshold level by ≥45% of the MDD participants. In contrast, the last item in Part A (number 6, feeling driven by a motor) was the item endorsed leased frequently by MDD participants, at 10%. The 55 HC participants rarely endorsed items at the threshold level; the most frequently endorsed item was number 16 (finishing the sentences of other people) at 5.5%.

### 3.3. ADHD Symptoms and Diagnosis by MINI

Based on the MINI ADHD module assessment, 20 participants (15 women and 5 men) met symptom count criteria for adult ADHD (16 inattentive type; 4 combined type). These 20 participants were all from the MDD patient group, indicating that 50% of MDD patients endorsed a sufficient number of current inattention symptoms to meet the symptom count criterion for ADHD. Of the 20 meeting the symptom count criterion, only 7 continued to meet full diagnostic criteria after application of criterion TA3 (onset of symptoms before age 7) and 2 of these did not meet the TA4 criterion of functional impairment, resulting in only 5 of the 40 MDD participants (12.5%) meeting the full DSM-IV diagnostic criteria for ADHD (2 inattentive and 3 combined type). The psychiatrists’ assessments did not change any of these participants’ ADHD diagnoses. We also examined the effect of applying the DSM-5 ADHD diagnosis threshold of ≥5 symptoms of a subtype (as opposed to ≥6 required for DSM-IV). Six additional MDD patients would have met the DSM-5 reduced symptom count criterion (2 inattentive subtype, 4 hyperactivity/impulsivity subtype), totaling 26/40 (65%) of the MDD sample.

Table 2 compares the 20 ADHD symptom-count criterion positive MDD participants versus the 20 MDD participants who did not meet the symptom count criterion. Notably, depression severity did not differ between the two groups of depressed patients. However, the patients who met ADHD symptom count criterion reported higher levels of anxiety as assessed by the HAMA.

For the 5 formal DSM-IV diagnosis ADHD patients, no significant differences emerged between them and the 35 other MDD patients on any of the categorical or continuous variables.

### 3.4. Utility of ASRS-v1.1 in Depressed Patients

**Figure 2:**
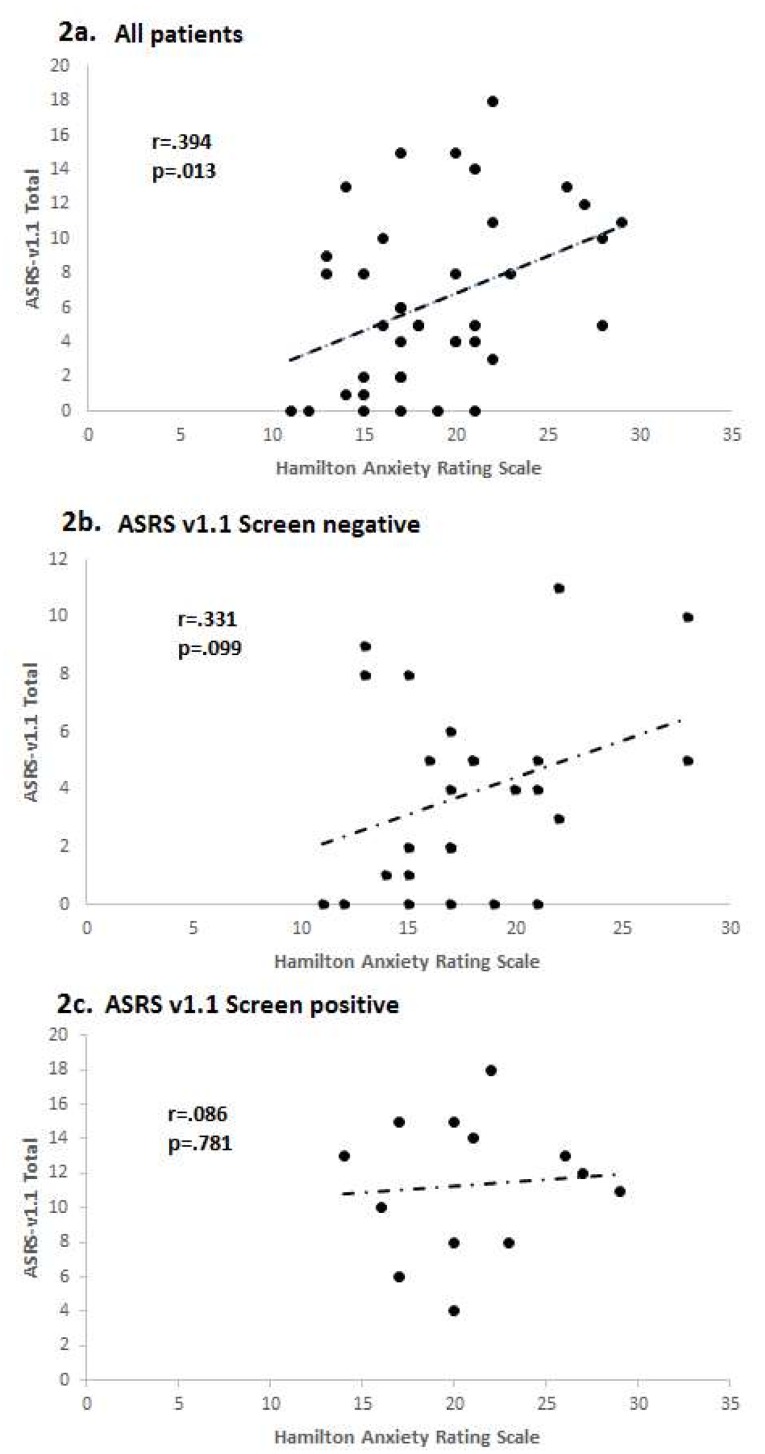
Correlations between anxiety and number of ASRS-v1.1 symptoms endorsed in depressed patients. (**a**) All depressed patients; (**b**) ASRS-v1.1 screen negative patients; (**c**) ASRS-v1.1 screen positive patients.

Fourteen of the 40 MDD patients screened positive on the ASRS-v1.1 Part A, with a score ≥4. As shown in Table 3, the screen positive patients had significantly higher anxiety (HAMA) scores. They also scored significantly higher on HAMA item 5 (Concentration impairment) and on the RRS. Among all 40 MDD patients, the number of ASRS-v1.1 items endorsed at the threshold level was positively correlated with anxiety (HAMA score), both for Part A (*r* = 0.336, *p* = 0.036) and for all 18 ASRS-v1.1 items (*r* = 0.394, *p* = 0.013) (Figure 2a). The correlation with anxiety was stronger for ASRS-v1.1 screen negative MDD patients (Figure 2b) than for screen positive patients (Figure 2c), though the association was not significant for either group.

Using the standard cut-off of ≥4 ASRS-v1.1 Part A items to indicate a positive ADHD screen, we found fair performance of the ASRS v1.1 as a screener in the MDD sample. Defining the 20 symptom-criteria level ADHD subjects as “cases” (i.e., ignoring the requirement for onset of symptoms before age 7 and functional impairment), the ASRS-v1.1 had a sensitivity of 55.0% (95% CI: 31.5–76.9%), specificity 85.0% (95% CI: 62.1–96.8%), PPV 78.6% (95% CI: 54.6–91.8%), NPV 65.4% (95% CI: 52.9–76.0%) and total classification accuracy 70% (95% CI: 53.5–83.4%).

When the cases were defined as the 5 participants who met the full DSM-IV ADHD criteria, including the age of onset and functional impairment criteria, the ASRS-v1.1 produced a substantially lower PPV and higher NPV, with similar total classification accuracy: sensitivity 60% (95% CI: 14.7–94.7%), specificity: 68.6% (95% CI: 50.7–83.2%), PPV 21.4% (95% CI: 10.3–39.4%), NPV 92.3% (95% CI: 80.0–97.3%) and total classification accuracy 67.5% (95% CI: 50.9–81.4%). All three full-syndrome combined type ADHD cases met the ASRS Part A threshold of ≥4. The two full-syndrome ADHD cases who did not meet the ASRS Part A threshold were both inattentive type and both scored 3 on Part A.

We also examined the differences in frequency of endorsing ASRS-v1.1 items positively between the patients who were false positives on Part A of the ASRS-v1.1 (*n* = 11) versus those who met full-syndrome criteria for ADHD (*n* = 5) (Table 4). Items 5 and 6 from Part A, along with items 10, 11, 13 and 17 from Part B, all of which identify hyperactivity and impulsivity symptoms, showed greater specificity for full syndrome ADHD.

## 4. Discussion

To our knowledge, this is the first study to report on the performance of the ASRS-v1.1 as a screening instrument for ADHD in adults with a primary MDD diagnosis. The prevalence of full criteria DSM-IV ADHD was 12.5% among the depressed sample, which is similar to prior estimates in community (9.4%) [4] and clinical MDD samples (8–14%) [9,13]. In identifying full criteria ADHD, the ASRS demonstrated fair sensitivity (60%) and specificity (68.6%), a low PPV (21.4%) and excellent NPV (92.3%), with total classification accuracy of 67.5%. When the definition of ADHD was broadened to include those patients who met only the *symptom count criterion* for inattentive, hyperactive-impulsive, or combined type (but not the DSM-IV age of onset or impairment criteria), sensitivity was similar but the specificity (85%), PPV (78.6%) were improved, though the NPV was reduced (65.4%), resulting in similar total classification accuracy (70%).

The high NPV of the ASRS-v1.1 is consistent with other studies examining the performance of the ASRS-v1.1 in patients with primary substance use disorders [18] and primary care settings [30,31]. Thus, the ASRS-v1.1 may have utility as a quick means for ruling out comorbid ADHD, because patients who test negative are unlikely to have the condition. However, it should be noted that two of the five true ADHD cases did not screen positive on the ASRS-v1.1, indicating that further evaluation for ADHD may be warranted if clinical suspicion is sufficiently high. A positive screen on the ASRS-v1.1 should always lead to careful subsequent evaluation by the clinician, as the positive predictive value is substantially lower (roughly 20–50%) [18,30]. Thus, a positive Part A screening score on the ASRS-v1.1 is more likely to be a false positive than a true positive in patients with mood or substance disorders. It is also notable that 50% of the MDD sample endorsed sufficient numbers of symptoms on the MINI interview to meet the symptom count criteria for ADHD, which was a higher positive rate than the 35% who screened positive using the ASRS-v1.1 Part A. Consequently, neither simple symptom counts nor ASRS-v1.1 screening can be considered sufficient in themselves for determining ADHD diagnosis and treatment. This conclusion is supported by a recently study of 239 individuals repeatedly assessed for ADHD and other psychiatric and substance used disorders from childhood through young adulthood [32]. Roughly 95% of the subjects in this study who screened positive for ADHD using symptom checklists were ultimately found not to have late-onset ADHD, largely as a result of applying Criterion E (i.e., symptoms or impairment occurred as part of a comorbid psychiatric disorder or heavy substance use) [32].

The high potential for false positives using Part A of the ASRS-v1.1 to screen for ADHD is demonstrated in that of the five most frequently endorsed 18 ASRS-v1.1 items, four were from the six Part A items and all of them were inattention items (items 1–4). The other two Part A items that refer to hyperactivity, including “feeling driven by a motor”, which was the least frequently endorsed of the 18 items.

Depressed patients screening positive on the ASRS-v1.1 reported significantly more anxiety and rumination than patients screening negative. Interestingly, neither anxiety nor rumination was associated with the ADHD symptoms as assessed by the structured MINI interview. Having any lifetime anxiety disorder was not associated with either ASRS-v1.1 or MINI ADHD symptom endorsement. These data suggest that anxious (compared to non-anxious) depression, defined as high levels of current anxiety symptoms is associated with greater subjective reports of inattention. One prior study found that anxious arousal but not anxious apprehension, was associated with greater cognitive impairments [33], which could explain this association. Whether anxious depression is more common among patients with comorbid ADHD has not previously been examined and warrants further study. We are unaware of any prior studies examining levels of rumination among patients with ADHD; given that patients with ADHD frequently experience mind-wandering, which may be conceptualized as the opposite of rumination, this result was unexpected [34].

Impairments in cognitive control, specifically impaired inhibition, contribute to both ruminative thinking and reduced emotion regulation [35,36] and provide a potential mechanism to link the observed association between subjective levels of rumination and inattention in our sample. The cognitive load incurred by rumination likely reduces processing resources available for application to external material [37,38], which manifests as impaired concentration or inattention. Another possibility is that highly ruminative patients, as a form of cognitive distortion, more extensively recall and emphasize their errors or limitations in cognition, resulting in high endorsement on self-reported measures of both rumination and inattention.

A recent important development in ADHD has been the emergence of epidemiologic evidence suggesting the existence of an adult-onset form of ADHD. Three large studies from New Zealand [7], the United Kingdom [39] and Brazil [40] reported rates of adult-onset ADHD of 2.7%, 5.5% and 10.3%, respectively. In each study, the prevalence of adult-onset ADHD was greater than the prevalence of childhood-onset ADHD, which has led to concerns that the studies, all of which relied solely on self-report data to diagnose adult-onset ADHD, may have biased estimates [41]. It is also possible that many of the identified adult-onset cases represented the delayed full expression of sub-syndromal cases of childhood ADHD [42]. Such patients would not qualify for ADHD as defined by the DSM but nevertheless may share a vulnerability to the disorder that becomes manifest through the accumulation of stressors and possibly reduced parental support, during young adult years. That the prevalence of adult-onset ADHD is not as high as the epidemiologic studies suggest is supported by data from a specialty ADHD evaluation clinic in France, which identified adult-onset ADHD in only 7% of 446 patients specifically referred for ADHD evaluation [42]. Furthermore, only 2.8% of this sample had isolated adult-onset ADHD without a comorbid disorder, further highlighting the importance of mood and anxiety disorders as contributors to self-reported ADHD symptoms [43]. However, in this study the timing of onset of ADHD symptoms was assessed using retrospective clinical interviews, which may have introduced a recall bias into the assessments; the aforementioned epidemiologic studies all incorporated prospective follow-up data from childhood, minimizing the effect of this bias.

Limitations to this analysis include the relatively small sample size of depressed patients. However, the 12.5% prevalence of full syndrome ADHD in our MDD sample is consistent with other prevalence data in MDD patients [4,9,13]. This result, along with the racial diversity of the participants, suggest that the ADHD characteristics of our sample can be considered to be reasonably representative of adults with MDD. Another limitation was that we did not have informant reports, such as teacher’s evaluations or parental input, which could have improved the veracity of our assessment of childhood ADHD symptoms [44]. Hence, it is possible that our results underestimated the true rate of ADHD in the sample and consequently underestimated the accuracy of the ASRS-v1.1 in MDD patients.

Recently, the World Health Organization published a new scale for detecting ADHD in adults applying the DSM-5 criteria, the Adult ADHD Self-Report Scale (ASRS) [45]. This scale was found to have excellent operating characteristics, with a sensitivity and specificity exceeding 90% and a PPV of 67–83%, depending on the sample. The improved performance may stem in part from the more relaxed criteria for diagnosing ADHD in adults using DSM-5, which has resulted in a near doubling of the prevalence of adult ADHD in the U.S. (from 4.4% with DSM-IV to 8.2% with DSM-5 criteria), thereby enhancing the PPV [40]. In considering these very strong measures of accuracy, it should be noted that DSM-5 criterion E (specifying that symptoms are not better accounted for by another mental disorder) was only “indirectly” evaluated in these assessments of the screening instrument, without application of formal structured assessments [45] (p. 522). Given the results in the current analysis of treatment-seeking MDD patients, the DSM-5 ASRS may face the same challenges as the ASRS-v1.1, specifically the high rate of false positives among patients with mood disorders.

## 5. Conclusions

In adult MDD patients, detecting comorbid ADHD is challenging due to the overlap in cognitive symptoms between the disorders. Applying the ASRS-v1.1 as a screener in these patients can help rule out the presence of ADHD when the screening result is negative. However, positive ASRS-v1.1 screen results are more likely to be false positives rather than true positives. Consequently, treatment for ADHD among MDD patients who screen positive on the ASRS-v1.1 should only be initiated after careful clinician evaluation of the time course of the ADHD symptoms and their relationship to major depressive episodes.

## Figures and Tables

**Table 1:** Proportion of the 40 Major Depressive Disorder (MDD) participants endorsing each ASRS-v1.1 item at a frequency that suggests the symptom is consistent with a diagnosis of Attention Deficit/ Hyperactivity Disorder (ADHD).

Item No.	Item Description	Number Endorsing	%
1	Trouble wrapping up final details of a project	25	62.5
9	Difficulty concentrating on what people say to you, even when speaking to you directly.	25	62.5
2	Difficulty getting things in order for a task requiring organization	24	60.0
4	Avoiding getting started on a task requiring a lot of thought	19	47.5
3	Problems remembering appointments or obligations	18	45.0
8	Difficulty keeping your attention when doing boring or repetitive work	17	42.5
16	Finishing sentences of people you are talking to before they can finish them themselves	17	42.5
10	Misplacing or having difficulty finding things at home or work	15	37.5
14	Difficulty unwinding and relaxing when you have time to yourself	15	37.5
5	Fidget or squirm with your hands or feet when you have to sit down for a long time	13	32.5
18	Interrupting others when they are busy	13	32.5
11	Distracted by activity or noise around you	12	30.0
13	Feel restless or fidgety	12	30.0
12	Leave your seat in meetings or situations in which you are expected to remain still	8	20.0
7	Make careless mistakes when you have to work on a boring or difficulty project	7	17.5
15	Find yourself talking too much when you are in social situations	7	17.5
17	Difficulty waiting your turn in situations when turn taking is required	6	15.0
6	Feel overly active and compelled to do things, like you were driven by a motor	4	10.0

**Table 2:** Comparison of MDD patients who do or do not meet the DSM-IV inattentive symptom count criterion for ADHD on the MINI.

	MINI ADHD Inattentive Symptom Count Criterion Negative (*n* = 20)		MINI ADHD Inattentive Symptom Count Criterion Positive (*n* = 20)			
Characteristic	Mean	SD	Mean	SD	*t*	*p*
Age (years)	49.4	8.4	49.7	7.9	0.14	0.89
Age at first episode (years)	27.1	15.5	33.3	16.0	1.19	0.24
Number of episodes	2.3	1.2	2.3	1.3	0.13	0.90
HDRS	20.1	3.2	21.3	3.9	1.03	0.31
CGI-S	4.2	0.6	4.3	0.6	0.53	0.60
HAMA	18.1	4.2	19.6	4.9	1.02	0.32
HAMA Item 5 (Concentration)	1.9	0.8	2.5	0.5	3.03	**0.004**
CTQ Total	66.9	17.7	67.2	12.2	0.05	0.96
RRS	56.0	10.0	60.1	10.3	1.13	0.227
ASRS-v1.1 Part A	1.9	1.5	3.3	1.8	2.79	**0.008**
ASRS-v1.1 Part B	2.2	2.2	5.6	3.7	3.54	**0.001**
ASRS-v1.1 Total	4.0	3.2	8.9	5.3	3.50	**0.001**
	**N**	**%**	**N**	**%**	**χ^2^**	***p***
Sex					0.13	0.72
Male	6	30	5	25		
Female	14	70	15	75		
Race *					4.58	0.10
White	12	67	7	35		
Black	5	28	8	40		
Other/Multiple	1	6	5	25		
Marital Status *					0.21	0.65
Married/Partnered	7	35	8	42		
Single	13	65	11	58		
Currently Employed					0.44	0.51
Yes	12	60	14	70		
No	8	40	6	30		
Lifetime Anxiety Disorder					0.40	0.53
Yes	10	50	8	42		
No	10	50	12	58		
Lifetime Tobacco Use					1.0	1.0
Yes	10	50	10	50		
No	10	50	10	50		
Lifetime Substance Abuse					1.76	0.19
Yes	5	25	9	45		
No	15	75	11	55		
History of Stimulant Rx					0.23	0.63
Yes	3	15	2	10		
No	17	85	2	90		

* Number of subjects is less than 40 due to missing data. Bolded values represent *p* < 0.05.

**Table 3:** Comparison of MDD patients who screened positive versus negative for ADHD on the ASRS-v1.1.

	ASRS-v1.1 Part A Screen Negative (*n* = 26)		ASRS-v1.1 Part A Screen Positive (*n* = 14)			
Characteristic	Mean	SD	Mean	SD	*t*	*p*
Age (years)	48.2	8.2	52.1	7.6	1.49	0.15
Age at first episode (years)	30.1	14.7	30.2	18.3	0.02	0.99
Number of episodes	2.3	1.2	2.4	1.4	0.26	0.80
HDRS	20.1	3.1	21.8	4.1	1.48	0.15
CGI-S	4.2	0.6	4.4	0.5	1.43	0.16
HAMA	17.8	4.3	20.9	4.5	2.14	**0.039**
HAMA Item 5 (Concentration)	2.0	0.7	2.5	0.5	2.57	**0.014**
CTQ Total	64.5	12.6	71.8	18.0	1.43	0.16
RRS	55.6	9.4	62.8	10.6	2.10	**0.042**
No. MINI Inattention items	4.1	2.3	6.8	2.5	3.42	**0.002**
No. MINI Hyperactivity items	1.9	1.7	3.7	2.3	2.86	**0.007**
	**N**	**%**	**N**	**%**	**χ^2^**	***p***
Sex					0.40	0.52
Male	8	31	3	21		
Female	18	69	11	79		
Race *					0.13	0.94
White	12	46	7	50		
Black	9	35	4	29		
Other/Multiple	4	15	2	14		
Marital Status *					0.05	0.49
Married/Partnered	9	35	6	46		
Single/Widowed/Divorced	17	65	7	54		
Currently Employed					0.39	0.53
Yes	16	62	10	71		
No	10	38	4	29		
Lifetime Anxiety Disorder					0.04	0.84
Yes	12	46	6	43		
No	14	54	8	57		
Lifetime Tobacco Use					0.44	0.51
Yes	12	46	8	57		
No	14	54	6	43		
Lifetime Substance Abuse					0.58	0.45
Yes	8	31	6	43		
No	18	69	8	57		
History of Stimulant Rx					5.09	**0.043**
Yes	1	4	4	29		
No	25	96	10	71		

* Number of subjects is less than 40 due to missing data; Bolded values represent *p* < 0.05.

**Table 4:** Frequency of positive endorsement of the six Part A ASRS-v1.1 items among MDD patients with a false positive ASRS-v1.1 Part A screen versus those with comorbid full syndrome ADHD.

Item No.	Item Description	False Positive ASRS-v1.1 Part A (*n* = 11)	Full Syndrome ADHD (*n* = 5)
1	Trouble wrapping up final details of a project	100%	100%
2	Difficulty getting things in order for a task requiring organization	100%	80%
3	Problems remembering appointments or obligations	91%	60%
4	Avoiding getting started on a task requiring a lot of thought	100%	80%
5	Fidget or squirm with your hands or feet when you have to sit down for a long time	27%	60%
6	Feel overly active and compelled to do things, like you were driven by a motor	18%	40%
